# High Throughput Phenotyping for Various Traits on Soybean Seeds Using Image Analysis

**DOI:** 10.3390/s20010248

**Published:** 2020-01-01

**Authors:** JeongHo BAEK, Eungyeong Lee, Nyunhee Kim, Song Lim Kim, Inchan Choi, Hyeonso Ji, Yong Suk Chung, Man-Soo Choi, Jung-Kyung Moon, Kyung-Hwan Kim

**Affiliations:** 1 National Institute of Agricultural Sciences, Rural Development Administration (RDA), Jeonju 54874, Korea; firstleon@korea.kr (J.B.); wowlek44@korea.kr (E.L.); knh702@korea.kr (N.K.); greenksl5405@korea.kr (S.L.K.); inchchoi@korea.kr (I.C.); jhs77@korea.kr (H.J.); moonjk2@korea.kr (J.-K.M.); 2 Faculty of Bioscience and Industry, College of Applied Life Science, SARI, Jeju National University, Jeju 63243, Korea; yschung@jejunu.ac.kr; 3 National Institute of Crop Sciences, Rural Development Administration (RDA), Wanju-gun 55365, Korea; mschoi73@korea.kr

**Keywords:** RGB image, seed morphology, seed color, seed traits, breeding

## Abstract

Data phenotyping traits on soybean seeds such as shape and color has been obscure because it is difficult to define them clearly. Further, it takes too much time and effort to have sufficient number of samplings especially length and width. These difficulties prevented seed morphology to be incorporated into efficient breeding program. Here, we propose methods for an image acquisition, a data processing, and analysis for the morphology and color of soybean seeds by high-throughput method using images analysis. As results, quantitative values for colors and various types of morphological traits could be screened to create a standard for subsequent evaluation of the genotype. Phenotyping method in the current study could define the morphology and color of soybean seeds in highly accurate and reliable manner. Further, this method enables the measurement and analysis of large amounts of plant seed phenotype data in a short time, which was not possible before. Fast and precise phenotype data obtained here may facilitate Genome Wide Association Study for the gene function analysis as well as for development of the elite varieties having desirable seed traits.

## 1. Introduction

Accurate measurement and definition on morphology and color measurements of seeds are very difficult to obtain because it is time consuming and requires effort even for small number of samples. Most importantly, it is almost impossible to have objective data sets for colors and shapes of seeds with conventional methods. To overcome these issues, recent plant-phenotypic studies using images have been conducted [1,2,3,4,5,6,7]. These studies are methods of acquiring the seed images, processing images and analyzing them, and identifying information related to phenotypes. In this regard, image analysis of high-throughput methods of plant traits such as plant structure and physiological state is recently developed as a new research area [8,9,10]. Here, we have performed research on measuring and analyzing the morphology and color of plant seeds using high-throughput method with imaging equipment. In order to establish the basis of soybean genome breeding in National Institute of Crop Science (NICS) of Rural Development Administration (RDA), 407 cultivars and 393 wild species were selected from 4382 populations to construct core group [11]. In the core group, the agronomically desirable traits such as the seed color, the leaf type, the middle and the flowering days were investigated through actual measurements, which requires a lot of time and effort. Therefore, we determined that we needed efficient measurement and analysis of large quantities of soybean seeds via high-throughput phenotyping. The purpose of the current study is to measure and analyze the various phenotypes in seeds of the soybean core population in order to be able to study the gene function analysis to develop the elite variety related to the agronomically desirable traits.

## 2. Materials and Methods

**Figure 1:**
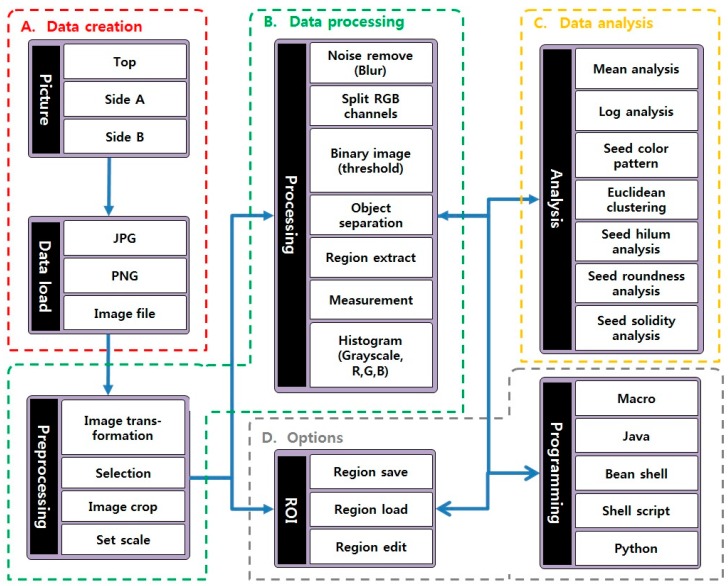
Flow-chart for high-throughput soybean seed phenotype analysis. The picture using raw image file is divided into four. (**A**) Data creation; (**B**) Data processing; (**C**) Data analysis; (**D**) Options.

The phenotypic analysis of soybean seeds was carried out using optical properties. One hundred soybean seeds were photographed at one time using a digital camera and the morphological and color indexes of each soybean seed were calculated through image processing. We also analyzed the characteristics of the data values on the measured results using the images. We devised a method for analyzing high-throughput method soybean seed phenotype using images to measure the phenotype of 39,065 seeds. And, we designed a phenotype analysis flow-chart as shown in Figure 1. The soybean seed phenotype high-throughput method is based on four efficient technical methods for data creating, processing, analysis and option.

### 2.1. Data Creation

**Figure 2:**
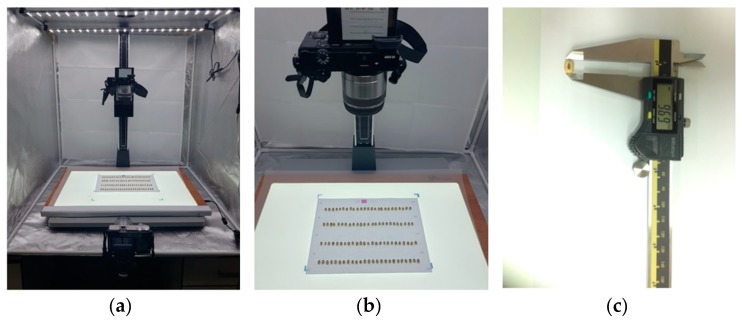
Device of soybean seed phenotype analysis used in high-throughput method. (**a**) top and side camera; (**b**) arrangement of soybean seeds; (**c**) actual measurement using Vernier calipers.

First, the data creation of the red block A in Figure 1 was performed to measure the basic size and thickness of each seed through the image taken at the top and the side. In order to calculate the data compared to the actual size, a tag of 16 mm in size was used as scale bar information. Also, to reduce the error of the color value depending on the light condition, a standard color was selected and a color tag was added. In order to reduce image errors caused by shadows during data production, the shadows of soybean seeds were removed using backlight and Light-Emitting Diode (LED) lighting. This method is one of the necessary methods to increase data reliability in analyzing the size and color of soybean seeds. Figure 2 shows the image capture settings for phenotyping environmental conditions. The camera view resolution was adjusted to the area of 210 × 297 mm, and 100 seeds were arranged in 4 lines of 25 each, and the images were taken with one top image and four side images. In order to check the correlation between image data and actual data, 9 lines randomly selected out of 400 lines were measured and compared using Vernier calipers.

### 2.2. Data Processing

Data processing of images taken using ImageJ program was performed. The data processing of the green block B in Figure 1 consists of pre-processing and post-processing. First, preprocessing consists of selection and transformation of images, cropping of size and color related tags, and setting of scale. Images were selected and transformed as needed in the shooting images and size and color related tags were removed for measurement and analysis of soybean seeds. To do this, we performed editing using area selection tools and macros. Also, in order to convert the size into mm in the scale setting, the standard tag is selected and set to the size of 16 mm for the pixel. After that, each soybean seed is separated and post-processing such as size measurement based on pixel value and color value extraction is performed. After preprocessing, separation of Red, Green, Blue (RGB) channel was performed to separate seeds and background using split channels function of ImageJ. The separation of seed and background is difficult to separate clearly due to the colorful color. However, the separation of RGB channels makes it easier to separate seeds and backgrounds by simplifying the color of the seeds according to color characteristics. After separation of RGB channels, binary images were created using threshold values of pixel values to complete separation of seeds and background. After that, we used the A function of ImageJ to measure the values of 100 seed areas, each of which separated the parts recognized as independent areas without connecting the objects. At this time, in order to exclude the noise which is not completely removed in the image processing process, the area smaller than the pixel size of 100, which is a smaller area than the soybean seed, is processed. The areas of each soybean seed thus treated were stored and managed in the Region of Interest (ROI) of ImageJ for the measurement and analysis of various indicators. Based on the stored ROI, we overlaid on the original image and measured various pixel-based information of the corresponding region. The measured information is Area, perimeter, width, height, thickness, circularity, aspect ratio, roundness, solidity and histogram (red, green, blue, grayscale), which are judged to be related to traits. In addition, image coordinate values of each bean were measured in order to match the top and the side image and to integrate the measured data from the image. These tasks should be performed for each line and image, but they are performed collectively using macros to reduce the cost of time and effort.

### 2.3. Data Analysis

We integrated the data measured in the image processing stage using python programming and analyzed the morphology and color relation as shown in orange box C in Figure 1. First, morphological analysis classified the differences among the total seeds using the values of each measured index. In this paper, we used the roundness index to detect the feature that the morphology of the bean seeds is different depending on the difference in length. Also, we used solidity index to classify defective soybean seeds whose appearance is not smooth. Using the maximum, minimum, and standard deviation, we classified the seeds by group, and clustering was used to find out the seeds that were obviously wrong in the corresponding soybean. Color analysis was performed to find peaks with a specific value within the mean value range of histogram of soybean seeds. In addition, the histograms at both ends are changed to very low values, and these parts are analyzed using a log which can be confirmed by correcting the distortion due to the data deviation. This color analysis gave us various results such as a specific trait presumed to be a virus infection and other colors of Hilum.

### 2.4. Options

It is too much time and effort for each individual to analyze 39,065 phenotypic analyzes of soybean seeds. Therefore, we took 100 seeds in one line in the data creation and processed the process and analysis through macro and programming like the gray block D in Figure 1. We used macros provided by ImageJ to process the image data. The measured data was saved in csv format and used python program. In order to recycle images according to new phenotypic analysis, region information of individual soybean seeds was saved as ROI.

## 3. Results

### 3.1. Detection of Specific Traits Using Morphology Analysis

**Figure 3:**
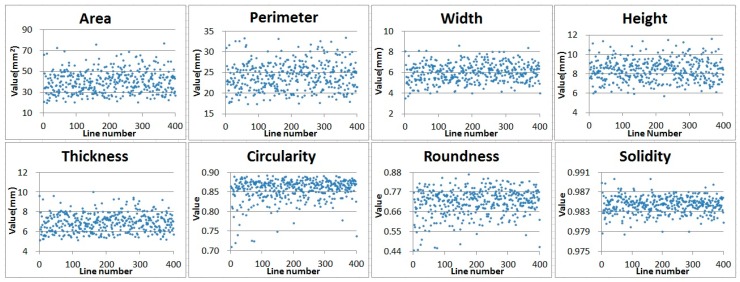
Distribution of morphological data measurement of 400 lines. It is a distribution chart that calculates the average area, perimeter, width, height, thickness, circularity, roundness, solidity of each lines.

We measured 8 kinds of morphological data such as area, perimeter, width, height, thickness, circularity, roundness, and solidity (Supplementary Materials, Table S1) [12] in total of 39,065 seeds of about 100 seeds in 400 cultivated soybean lines using image based high-throughput method (Supplementary Materials, Table S2). The measured data were calculated by the average of each cultivar and the results as shown in Figure 3 were obtained. Overall, some were evenly distributed across classes, while others were not. The distribution of phenotype from the precise measurement by image analysis shows those traits seems to be quantitative values as shown in Table 1. Each morphological data was divided into 5 classes with a constant interval based on Table 2.

**Figure 4:**
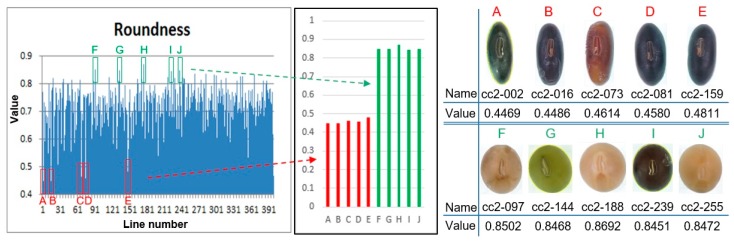
Classification of morphological phenotype data of the Roundness. A red box (A, B, C, D, E) with an elongated shape has a roundness value of 0.4~0.5 and a green box (F, G, H, I, J) with a close to a circle shape has a roundness value of 0.8 or more.

The eight morphological phenotype data thus measured can be used to compare and analyze agricultural traits between lines [13]. It can also be used as very useful data for detecting individuals with specific traits in varieties. We confirmed the roundness calculated by the ratio of the lines size in the measured data as shown in Figure 3, and it was able to confirm several kinds of varieties information having a relatively different value. As a result, we did morphological classify to the roundness according to the ratio of varieties based on the interval values as shown in Figure 4. Most lines have roundness distribution between 0.6~0.7, but A, B, C, D, E have a value of 0.4~0.5 and are classified as having relatively elongated proportions. Also, F, G, H, I, and J were classified as cultivars with a ratio of 0.8 or more and a relatively close circle.

**Figure 5:**
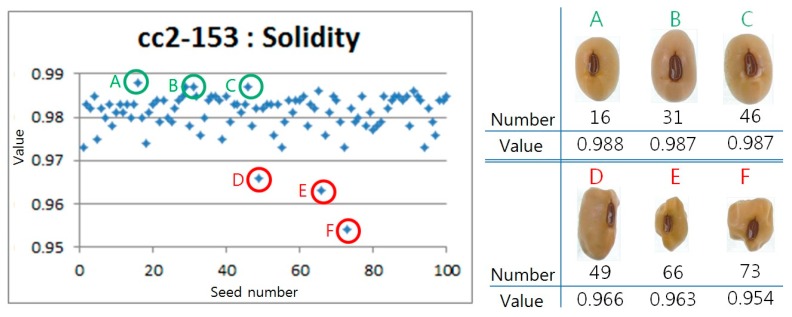
Detecting specific morphology using the Solidity. Good seeds are A, B, C and bad seeds are D, E, F.

In addition, using solidity values representing the smoothness of the surface, we also detected soybean seeds with specific morphology as shown in Figure 5. In 100 seeds of the cc2-153 line used in the experiment, most of the normal soybean seeds such as A, B and C have solidity values between 0.97 and 0.99. We determined that the deviation from this distribution was the damage caused by *Pentatomidae* and detected three D, E, F seeds with solidity values of less than 0.97. According to the results, it was possible to detect seeds showing a specific morphology.

### 3.2. Detection of Specific Traits using Color Analysis

**Figure 6:**
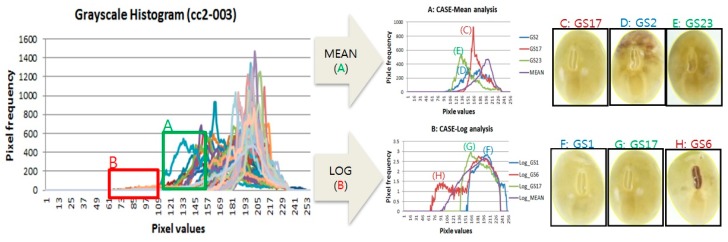
Grayscale graph analysis of cc2-003 line. A green box A shows the results C, D, E using the mean value and a red box B shows the results F, G, H using the mean value.

Information on the color of soybean seed is very important because it relates to various traits such as functional substances, diseases, and the environment [14,15,16]. Therefore, we analyzed color data of RGB and grayscale channels for each 39,065 seeds and analyzed it by using pixel distribution graph. Red, green, and blue histograms were used to classify soybean seeds color, and grayscale histograms were used to detect features with distinct patterns [17]. Figure 6 shows graphs of the average values and the values of each object according to the seed brightness of the cc2-003 line using grayscale. As a result, a red box A and a green box B outside the range of the average value graph were found. Then, only the corresponding seeds were extracted and the graph was redrawn. As a result, each seed image was confirmed as shown in Figure 6. The green box B was analyzed using the average value and soybean seeds with specific colors such as D and F were detected based on the C seeds which are seen in general color. In addition, a graph drawn by a very small value such as red box A was analyzed using the log value. As a result, F and G were normal soybeans and H was analyzed as soybeans having a specific pattern of hilum color.

**Figure 7:**
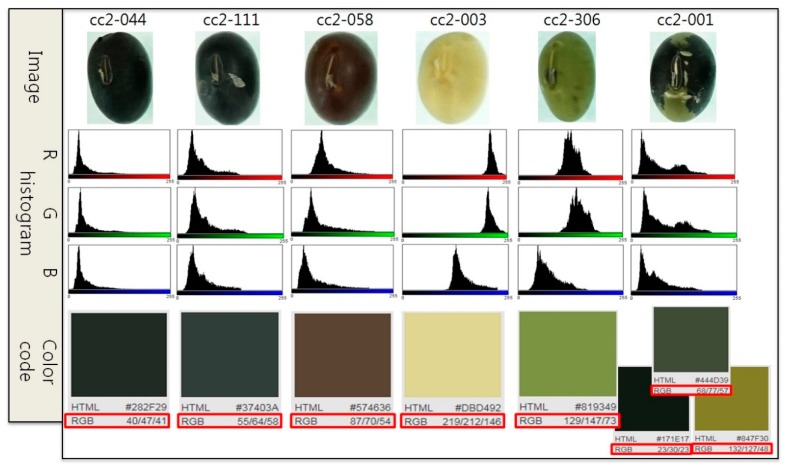
Quantitative color classifications of soybean seed using Red, Green, Blue (RGB) histogram. The figure shows each line that the seed image, the RGB histogram, and the color code of red box.

We used python program to analyze of all soybean seeds in the same method as in Figure 6 and to detect 597 seeds with specific colors. We also analyzed the micro graph using log values and detected 929 specific colors. However, micro graphs show inaccurate values for soybeans that have overall dark color. Thus, the soybeans with bright colors were reanalyzed to detect specific color seeds having 138 different hilum colors. We also detected 389 seeds with duplicate results for mean value analysis. In addition, we quantitatively classify the color of soybean seeds by RGB channel analysis. Figure 7 shows the RGB histograms for five seeds (cc2-044, cc2-111, cc2-058, cc2-003, cc2-306) with one color and one seed (cc2-001) with two colors as shown in the figure. In the histogram result, representative values are specified according to the RGB color code used in the HTML code provided by the Web. This method is a method to more precisely and accurately divide the color classification of soybean seeds by quantified color values.

## 4. Discussion

The ability to be able to deal with large same kinds and numbers in an accurate manner are crucial to increase the discrimination power of an identification system because they depend not only on the intra-specific representativeness of the analyzed samples but also on the quality and quantity of the parameters measured and used to discriminate among groups, as well as on the dimension and variability degree of the groups according to recent papers [18,19]. Even the relatively recent study on soybean seed character had only small number of samples due to the difficulty as mentioned above [20,21].

**Figure 8:**
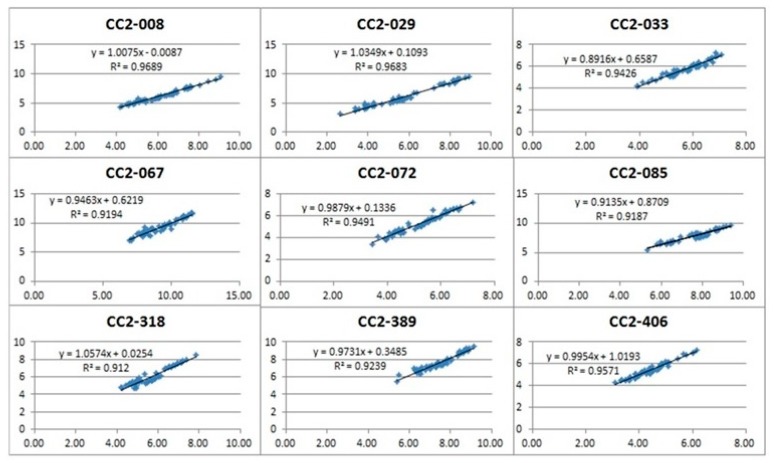
Correlation between actual measurement and image measurement of randomly selected 9 lines.

Here, we used Analyze particles function of ImageJ [22] to extract the data of each soybean seed from 100 seeds out of one frame of image. Overall, we could screen 39,065 seeds in automated and precise manner, which makes this method high throughout compared to conventional methods. Further, 9 lines randomly selected from 400 lines were actually measured for width, height, and thickness using Vernier Calipers. As a result of comparing the actual measured data with the data measured in the image, it was confirmed that it has a high correlation of about 0.94 as Figure 8.

## 5. Conclusions

In the current paper, we present a method of precisely investigating individual phenotypic information on various types of morphology and color in soybean seeds using high-throughput and an analysis method as the purpose of this study. As results, this method enables the investigation of large numbers of samples that are difficult for humans to measure and analyze the phenotypic data of plants that are time-consuming and labor intensive. We could rapidly measure and analyze about 39,065 soybean seeds of 400 lines in the proposed method. There have been no studies to deal with such number for seed phenotyping in this much accuracy. Furthermore, we opened new window for the seed color data collection which was very difficult to measure in an objective manner. These results could be applied to various analysis for shapes which are well addressed by Bianco et al. [23] and Cervantes et al. [24] for seed traits. Eventually, the accurate phenotypic data produced using the method of analysis presented in this research will produce would help to search the gene locus for the target through Genome Wide Association Study (GWAS). It could also be used as a method for precise phenotyping and analysis of many other plant seeds.

## Figures and Tables

**Table 1:** Distribution of the morphological data in the 400 lines.

**Area (mm^2^)**		**Perimeter (mm)**		**Width (mm)**		**Height (mm)**	
Class	Count	Class	Count	Class	Count	Class	Count
<10	0	<15	0	<2	0	<4	0
30	76	20	45	4	2	6	2
50	256	25	193	6	193	8	142
70	65	30	146	8	197	10	224
90	3	35	16	10	8	12	32
**Thickness (mm)**		**Circular**		**Roundness**		**Solidity**	
Class	Count	Class	Count	Class	Count	Class	Count
<4	0	<0.70	0	<0.44	0	<0.975	0
6	76	0.75	7	0.55	12	0.979	1
8	278	0.80	6	0.66	67	0.983	69
10	45	0.85	97	0.77	236	0.987	314
12	1	0.90	290	0.88	85	0.991	16

**Table 2:** Soybean 400 lines morphological range of measurements.

	Area	Perimeter	Width	Height	Thickness	Circular	Roundness	Solidity
Minimum	19.718	17.436	3.440	5.720	5.005	0.709	0.447	0.978
Maximum	77.103	41.741	8.527	11.654	9.854	0.892	0.869	0.990
Average	40.319	24.245	5.986	8.431	6.814	0.856	0.714	0.984
Stdev.	10.684	3.405	0.901	1.113	0.922	0.028	0.073	0.002

## Supplementary Materials

The following are available online at https://www.mdpi.com/1424-8220/20/1/248/s1, Table S1: Measurement feature of ImageJ used in this study, Table S2: Morphological data of soybean seeds in the 400 lines.
