# Supplementary material for: High Throughput Phenotyping for Various Traits on Soybean Seeds Using Image Analysis
Source: Sensors (Basel). 2020 Jan 1;20(1):248. doi: 10.3390/s20010248 (PMC6982885; doi:10.3390/s20010248)
Supplement: Supplementary file 1 [file sensors-20-00248-s001.pdf]

High Throughput Phenotyping for Various Traits on Soybean Seeds Using Image Analysis

JeongHo BAEK [1,‡], Eungyeong Lee [1,‡], Nyunhee Kim [1], Song Lim Kim [1], Inchan Choi [1], Hyeonso Ji [1], Yong Suk Chung [2], Man-Soo Choi [3], Jung-Kyung Moon [1] and Kyung-Hwan Kim [1,*]

[1] National Institute of Agricultural Sciences, Rural Development Administration (RDA), Jeonju, 54874, Korea; firstleon@korea.kr (J.B.); wowlek44@korea.kr (E.L.); knh702@korea.kr (N.K.); greenksl5405@korea.kr (S.L.K.); inchchoi@korea.kr (I.C.); jhs77@korea.kr (H.J.); moonjk2@korea.kr (J.-K.M.); biopiakim@korea.kr (K.-H.K.)

[2] Faculty of Bioscience and Industry, College of Applied Life Science, SARI, Jeju National University, Jeju, 63243, Korea; yschung@jejunu.ac.kr

[3] National Institute of Crop Sciences, Rural Development Administration (RDA), Wanju-gun, 55365, Korea; mschoi73@korea.kr

* Correspondence: biopiakim@korea.kr; Tel.: +82-63-238-4658

‡ These authors also contributed equally to this work.

Table S1. Measurement Feature of ImageJ used in this study.

Area	Area of selection in square pixels or in calibrated square units.
Perimeter	The length of the outside boundary of the selection.
Width, Height, Thickness	Fits an ellipse to the selection. best fitting elipse.
Circular	with a value of 1:0 indicating a perfect circle.
Roundness	the inverse of the aspect ratio of the particle's fitted ellipse.
Solidity	Convex Hull command makes an area? selection convex.

Table S2. Morphological data of soybean seeds in the 400 lines

No	Name	Area (mm2)	Perim. (mm)	Width (mm)	Height (mm)	Thickness (mm)	Circular	Roundness	Solidity
1	cc2-001	66.3678	31.0463	8.0458	10.4894	9.5026	0.8646	0.7683	0.9889
2	cc2-002	20.8880	19.1881	3.4398	7.7007	5.0054	0.7095	0.4469	0.9787
3	cc2-003	48.4282	26.5636	6.7054	9.1874	7.7893	0.8609	0.7319	0.9870
4	cc2-004	33.9017	22.8562	5.0266	8.5617	6.1889	0.8132	0.5899	0.9829
5	cc2-005	33.9438	22.2386	5.5526	7.7679	6.2805	0.8602	0.7161	0.9862
6	cc2-006	34.7982	23.2222	5.0471	8.7605	5.6684	0.8084	0.5775	0.9834
7	cc2-007	45.5548	25.8473	6.2924	9.2076	7.3988	0.8555	0.6850	0.9847
8	cc2-008	29.0264	20.7420	4.9159	7.4984	5.8931	0.8449	0.6580	0.9829
9	cc2-009	35.1069	22.7418	5.4893	8.1280	6.5599	0.8510	0.6775	0.9834
10	cc2-010	19.7185	17.7230	3.7051	6.7674	5.1557	0.7871	0.5488	0.9816

11	cc2-011	67.4249	31.6873	7.6359	11.2318	9.1332	0.8427	0.6811	0.9887
12	cc2-012	22.8722	17.9478	4.8112	6.0349	5.1152	0.8892	0.7975	0.9818
13	cc2-013	34.8296	22.7841	5.3006	8.3407	5.5316	0.8403	0.6379	0.9837
14	cc2-015	43.5391	25.0157	6.3601	8.6876	6.8614	0.8711	0.7338	0.9859
15	cc2-016	28.1068	22.1025	3.9930	8.9362	5.6212	0.7200	0.4486	0.9807
16	cc2-017	39.6867	23.7763	6.2453	8.0741	6.3408	0.8795	0.7753	0.9845
17	cc2-018	22.2228	17.9759	4.5330	6.2285	5.4069	0.8622	0.7287	0.9829
18	cc2-019	38.6301	23.6628	6.2015	7.9228	6.9287	0.8656	0.7839	0.9853
19	cc2-020	24.9626	18.8218	5.0835	6.2336	5.5152	0.8822	0.8180	0.9835
20	cc2-021	43.9649	26.1878	5.6501	9.8893	6.8668	0.8036	0.5737	0.9845
21	cc2-022	47.2767	26.3566	6.3856	9.4033	7.3549	0.8525	0.6829	0.9851
22	cc2-024	36.2852	22.9861	5.6820	8.1172	6.2934	0.8608	0.7034	0.9848
23	cc2-025	29.2636	22.2701	4.2091	8.8296	5.8038	0.7395	0.4775	0.9827
24	cc2-026	41.9187	24.7310	6.4490	8.2398	7.4750	0.8577	0.7845	0.9836
25	cc2-027	35.2446	22.5865	5.8416	7.6549	6.9034	0.8641	0.7654	0.9850
26	cc2-028	50.3336	27.0814	6.7780	9.4304	7.3645	0.8589	0.7216	0.9845
27	cc2-029	28.1292	21.4400	4.2475	8.4075	5.4706	0.7659	0.5066	0.9809
28	cc2-030	39.2981	23.7418	6.0808	8.2106	6.9447	0.8738	0.7417	0.9852
29	cc2-031	32.3862	21.7253	5.3702	7.6580	6.0292	0.8592	0.7030	0.9828
30	cc2-032	52.1941	32.6683	6.4261	10.3068	8.0691	0.8171	0.6260	0.9855
31	cc2-033	27.4506	19.7112	5.2107	6.6833	5.9315	0.8833	0.7803	0.9832
32	cc2-034	47.3669	26.0386	6.7804	8.8823	7.5456	0.8762	0.7655	0.9863
33	cc2-035	31.2740	21.9957	4.8023	8.2843	6.2594	0.8108	0.5811	0.9833
34	cc2-036	44.3100	25.2479	6.4685	8.7018	7.7521	0.8709	0.7460	0.9855
35	cc2-037	27.3726	19.6087	5.2759	6.5638	5.9536	0.8883	0.8043	0.9822
36	cc2-038	45.8943	25.8001	6.4904	8.9976	7.2857	0.8637	0.7258	0.9854
37	cc2-039	36.1152	22.6123	6.1008	7.5055	7.1521	0.8831	0.8158	0.9839
38	cc2-040	25.9989	20.2085	4.2756	7.6929	6.0103	0.7941	0.5572	0.9839
39	cc2-041	22.4112	18.4543	4.1757	6.8231	5.5867	0.8250	0.6135	0.9838
40	cc2-042	36.5746	23.1219	5.7151	8.1403	6.0363	0.8583	0.7060	0.9841
41	cc2-043	30.7970	21.1098	5.3050	7.3757	6.4353	0.8660	0.7205	0.9835
42	cc2-044	72.5982	32.6627	8.0853	11.4245	9.4108	0.8538	0.7104	0.9896
43	cc2-045	43.3595	25.1600	6.3754	8.6484	7.5422	0.8591	0.7386	0.9862
44	cc2-046	27.2275	19.6046	5.2489	6.5844	5.5506	0.8871	0.7996	0.9826
45	cc2-047	46.6578	25.7095	6.8793	8.6189	7.4313	0.8846	0.8006	0.9863
46	cc2-049	32.4984	21.5335	5.5053	7.4606	5.8255	0.8727	0.7371	0.9836
47	cc2-050	29.0345	20.3633	5.2721	7.0019	5.8055	0.8777	0.7560	0.9831
48	cc2-052	30.9217	21.0813	5.3663	7.3242	6.0128	0.8722	0.7340	0.9840
49	cc2-053	39.5437	24.2561	5.7594	8.7282	6.7332	0.8426	0.6621	0.9841
50	cc2-054	36.0652	23.8839	5.0324	9.1137	5.8103	0.7914	0.5549	0.9826

51	cc2-055	35.6889	22.4146	6.0621	7.4756	6.4818	0.8897	0.8118	0.9851
52	cc2-056	51.4211	27.1566	7.1193	9.1809	7.6047	0.8743	0.7776	0.9862
53	cc2-057	40.8078	24.4186	5.9941	8.6455	6.4631	0.8572	0.6952	0.9851
54	cc2-058	45.4389	25.6539	6.5150	8.8675	7.5802	0.8659	0.7355	0.9877
55	cc2-059	49.1386	26.9599	6.4420	9.6756	7.4879	0.8453	0.6751	0.9853
56	cc2-060	45.3906	26.5850	5.7945	9.9542	6.8417	0.8046	0.5855	0.9863
57	cc2-061	56.7179	33.3576	7.4412	9.6931	8.0051	0.8648	0.7688	0.9869
58	cc2-062	37.2019	23.7119	5.4569	8.6734	6.9872	0.8302	0.6309	0.9861
59	cc2-063	33.2400	22.2879	5.2408	8.0682	6.3244	0.8399	0.6512	0.9862
60	cc2-064	24.1955	19.0993	4.3903	7.0020	5.5513	0.8313	0.6280	0.9833
61	cc2-065	49.9594	31.2480	6.8022	9.3231	7.5864	0.8568	0.7312	0.9862
62	cc2-066	45.6914	25.6086	6.7329	8.6391	7.7965	0.8749	0.7810	0.9874
63	cc2-067	69.4301	31.6415	8.1385	10.8458	8.8888	0.8693	0.7529	0.9878
64	cc2-068	51.5383	27.1322	7.1285	9.1865	7.0458	0.8770	0.7768	0.9859
65	cc2-069	49.7914	26.6067	7.1581	8.8419	7.9927	0.8822	0.8118	0.9866
66	cc2-070	31.0079	21.8945	4.8509	8.1296	6.4218	0.8118	0.5978	0.9850
67	cc2-072	21.0508	17.4843	4.3232	6.1805	5.2105	0.8611	0.6997	0.9807
68	cc2-073	35.3845	24.6987	4.5424	9.8825	6.2792	0.7259	0.4614	0.9812
69	cc2-074	39.7513	27.3943	5.8916	8.5802	6.7878	0.8419	0.6923	0.9837
70	cc2-075	51.9648	27.7706	6.6169	9.9755	7.9580	0.8437	0.6651	0.9855
71	cc2-076	29.9421	20.6684	5.3647	7.0871	5.8670	0.8776	0.7587	0.9833
72	cc2-077	29.9458	20.7142	5.3328	7.1354	5.4803	0.8744	0.7495	0.9820
73	cc2-078	50.3801	27.8152	6.1807	10.3595	7.8858	0.8162	0.5979	0.9856
74	cc2-080	45.6248	25.6314	6.6073	8.7793	7.0994	0.8703	0.7561	0.9848
75	cc2-081	26.4216	21.3608	3.9175	8.5669	5.6577	0.7241	0.4580	0.9785
76	cc2-082	37.7182	23.2561	5.9763	8.0152	6.4613	0.8728	0.7473	0.9850
77	cc2-083	55.7494	28.2889	7.3064	9.7028	7.7164	0.8734	0.7546	0.9869
78	cc2-084	58.7841	29.1159	7.4249	10.0680	8.0075	0.8695	0.7387	0.9861
79	cc2-085	46.9030	25.9100	6.7279	8.8596	7.8338	0.8758	0.7614	0.9860
80	cc2-086	38.6375	23.5125	6.1002	8.0071	6.8893	0.8722	0.7635	0.9846
81	cc2-087	45.8805	26.0279	6.4755	9.0063	7.6159	0.8493	0.7205	0.9853
82	cc2-088	48.7348	26.6059	6.7010	9.2324	7.7212	0.8613	0.7299	0.9851
83	cc2-089	41.8171	24.4617	6.3741	8.3396	6.9150	0.8762	0.7672	0.9853
84	cc2-090	25.8650	19.5422	4.7791	6.8782	5.8895	0.8492	0.6962	0.9829
85	cc2-091	46.8696	26.1042	6.5892	9.0430	7.5605	0.8628	0.7311	0.9849
86	cc2-092	28.4423	20.8587	4.6389	7.7841	5.5352	0.8180	0.5968	0.9828
87	cc2-093	36.3383	23.2597	5.4704	8.4366	6.5743	0.8410	0.6502	0.9842
88	cc2-094	27.6435	19.7212	5.3263	6.5905	5.5745	0.8903	0.8095	0.9831
89	cc2-095	44.9786	25.3190	6.6123	8.6301	7.3694	0.8780	0.7706	0.9857
90	cc2-096	34.7314	22.5904	5.4615	8.0833	6.4988	0.8533	0.6765	0.9845

91	cc2-097	48.0166	26.2330	7.1998	8.4776	7.8307	0.8751	0.8502	0.9844
92	cc2-098	36.4583	22.7843	5.9976	7.7064	6.5093	0.8781	0.7784	0.9845
93	cc2-099	28.7064	20.7122	4.7900	7.5903	5.4648	0.8355	0.6331	0.9818
94	cc2-101	55.9077	28.5263	7.1378	9.9580	8.5370	0.8612	0.7195	0.9861
95	cc2-102	20.4673	17.4359	4.0972	6.3441	5.4184	0.8427	0.6482	0.9799
96	cc2-103	40.4390	24.1380	6.0940	8.4033	6.6301	0.8652	0.7257	0.9852
97	cc2-104	39.3529	23.7118	6.1328	8.1245	6.6461	0.8736	0.7550	0.9850
98	cc2-105	35.4867	22.9282	5.4306	8.3030	6.0566	0.8460	0.6557	0.9844
99	cc2-106	38.4542	23.6394	5.8565	8.3447	6.7056	0.8622	0.7034	0.9852
100	cc2-108	29.9805	20.8164	5.3365	7.1396	6.2997	0.8671	0.7493	0.9845
101	cc2-109	27.4014	20.3563	4.5960	7.5553	5.7968	0.8263	0.6094	0.9828
102	cc2-111	42.7161	25.4498	6.1093	8.8968	7.5525	0.8286	0.6884	0.9852
103	cc2-112	45.9252	26.3022	6.0901	9.5403	7.5907	0.8289	0.6413	0.9839
104	cc2-113	27.2948	20.2151	4.6853	7.4005	5.6600	0.8370	0.6355	0.9825
105	cc2-114	47.3384	26.1619	6.7458	8.9287	7.6587	0.8682	0.7570	0.9877
106	cc2-115	42.6441	24.7486	6.3513	8.5270	6.8131	0.8719	0.7468	0.9849
107	cc2-116	45.6980	26.1434	6.1685	9.4172	7.2653	0.8383	0.6571	0.9874
108	cc2-118	31.1054	21.0939	5.4455	7.2520	5.9538	0.8753	0.7537	0.9839
109	cc2-119	35.0641	22.5397	5.6427	7.9046	6.4967	0.8658	0.7163	0.9847
110	cc2-120	37.8044	23.3969	5.8776	8.1438	7.0133	0.8621	0.7231	0.9840
111	cc2-121	50.1776	27.1132	6.6351	9.6056	7.7057	0.8552	0.6931	0.9862
112	cc2-122	28.8837	20.3794	5.1657	7.1025	5.3669	0.8713	0.7281	0.9825
113	cc2-123	47.5513	26.7183	6.3361	9.5431	7.2952	0.8372	0.6683	0.9850
114	cc2-124	31.2694	21.5862	5.0268	7.8847	5.9433	0.8391	0.6442	0.9835
115	cc2-125	46.0787	26.6854	5.8359	10.0411	7.1662	0.8121	0.5822	0.9853
116	cc2-126	24.0017	18.3294	4.9948	6.0803	5.0524	0.8916	0.8234	0.9813
117	cc2-127	33.9564	22.5120	5.5328	7.8039	6.4404	0.8412	0.7127	0.9842
118	cc2-128	28.4769	20.0888	5.2934	6.8243	5.7582	0.8829	0.7767	0.9835
119	cc2-129	32.3229	21.5396	5.5290	7.4174	6.3039	0.8715	0.7460	0.9835
120	cc2-130	33.2337	21.9169	5.4971	7.6878	6.1812	0.8677	0.7158	0.9829
121	cc2-131	45.8811	25.6407	6.6840	8.7206	6.8438	0.8747	0.7683	0.9848
122	cc2-132	34.5468	22.3291	5.6019	7.8314	6.5661	0.8679	0.7165	0.9845
123	cc2-133	37.9473	24.2365	5.2864	9.1354	6.3661	0.8110	0.5809	0.9848
124	cc2-134	40.8594	24.2730	6.1230	8.4547	6.7669	0.8659	0.7266	0.9855
125	cc2-135	43.4948	25.1840	6.1596	8.9282	7.1844	0.8549	0.6977	0.9850
126	cc2-136	43.6653	24.9397	6.5834	8.4274	7.1507	0.8794	0.7834	0.9857
127	cc2-137	49.2789	26.5780	6.8614	9.1232	7.4033	0.8738	0.7556	0.9858
128	cc2-138	61.5261	30.2296	7.2133	10.8293	8.1733	0.8422	0.6696	0.9863
129	cc2-139	34.8498	23.4208	4.9660	8.9263	5.9486	0.7963	0.5616	0.9831
130	cc2-140	50.5863	27.4838	6.4179	9.9974	7.9504	0.8374	0.6440	0.9862

131	cc2-141	51.1681	27.3047	6.8761	9.4558	7.7939	0.8605	0.7284	0.9848
132	cc2-142	26.7132	22.4463	5.2405	6.4783	5.4439	0.8797	0.8099	0.9824
133	cc2-143	41.5179	24.4216	6.2311	8.4537	6.8122	0.8712	0.7384	0.9850
134	cc2-144	23.8804	18.3305	5.0657	5.9877	5.8787	0.8906	0.8468	0.9841
135	cc2-145	33.0995	22.0809	5.3333	7.9047	6.1883	0.8522	0.6790	0.9840
136	cc2-147	52.2631	27.4602	6.9756	9.5186	7.9366	0.8679	0.7360	0.9865
137	cc2-148	40.0507	24.0825	6.0726	8.3779	6.7510	0.8647	0.7264	0.9848
138	cc2-149	33.0362	21.8785	5.4750	7.6701	6.4860	0.8651	0.7144	0.9845
139	cc2-150	34.5938	22.2772	5.7266	7.6651	6.6316	0.8726	0.7483	0.9837
140	cc2-151	43.4545	25.2482	6.1790	8.9395	7.1432	0.8547	0.6942	0.9849
141	cc2-152	37.0539	23.6046	5.4136	8.6885	5.9328	0.8331	0.6248	0.9836
142	cc2-153	39.1638	24.4640	5.5822	8.9124	6.4657	0.8190	0.6297	0.9809
143	cc2-154	40.7307	24.0402	6.4078	8.0825	6.6738	0.8840	0.7944	0.9845
144	cc2-155	34.3526	22.3147	5.5567	7.8323	6.4566	0.8610	0.7117	0.9840
145	cc2-156	47.4859	26.5246	6.5049	9.2864	7.7148	0.8472	0.7042	0.9841
146	cc2-157	34.7169	22.7176	5.5000	8.0220	7.0226	0.8430	0.6886	0.9845
147	cc2-158	40.5939	24.6131	5.8024	8.8930	6.7258	0.8401	0.6546	0.9852
148	cc2-159	28.4810	21.8303	4.1624	8.6939	5.6323	0.7489	0.4811	0.9819
149	cc2-160	24.3417	19.5196	4.1681	7.4198	5.4566	0.8003	0.5637	0.9814
150	cc2-161	51.7090	27.3411	6.9226	9.4731	7.2007	0.8656	0.7328	0.9856
151	cc2-162	31.9885	21.2683	5.6177	7.2126	6.2265	0.8827	0.7798	0.9844
152	cc2-163	28.7307	20.5413	4.9686	7.3495	5.9243	0.8536	0.6780	0.9826
153	cc2-164	56.7750	28.9596	6.9818	10.3313	7.7508	0.8481	0.6785	0.9863
154	cc2-166	34.9760	26.9034	5.1528	8.6117	5.7429	0.8089	0.5996	0.9835
155	cc2-167	33.7832	22.2925	5.4517	7.8731	6.1161	0.8516	0.6945	0.9819
156	cc2-168	30.1856	21.1879	5.1726	7.4184	6.5529	0.8433	0.6990	0.9836
157	cc2-169	29.1571	21.0209	4.7572	7.7772	5.8705	0.8256	0.6129	0.9830
158	cc2-170	31.5694	21.7076	5.1234	7.7989	6.2119	0.8377	0.6606	0.9822
159	cc2-171	29.2601	20.5235	5.2085	7.1355	5.7507	0.8703	0.7307	0.9834
160	cc2-173	75.9308	33.1852	8.5266	11.3249	9.8542	0.8651	0.7547	0.9897
161	cc2-174	38.7810	23.7721	5.8793	8.3848	6.4721	0.8603	0.7023	0.9850
162	cc2-175	42.1417	25.1398	5.8777	9.0937	6.9603	0.8339	0.6497	0.9838
163	cc2-176	27.7265	19.8864	5.1858	6.7977	5.7360	0.8794	0.7640	0.9834
164	cc2-177	41.9474	24.3806	6.5888	8.0929	7.5820	0.8850	0.8144	0.9875
165	cc2-178	52.3387	27.2177	7.2675	9.1041	7.7542	0.8809	0.7992	0.9862
166	cc2-179	57.2217	28.4950	7.6267	9.5358	8.5999	0.8833	0.8015	0.9870
167	cc2-180	46.0304	26.0894	6.3840	9.1561	6.9491	0.8478	0.6985	0.9845
168	cc2-181	38.2398	23.4085	6.0930	7.9775	6.8545	0.8747	0.7666	0.9847
169	cc2-182	62.2176	30.2548	7.4344	10.6419	8.2917	0.8526	0.7013	0.9865
170	cc2-183	30.3431	20.7499	5.4394	7.0711	5.5957	0.8807	0.7709	0.9820

171	cc2-184	44.4809	25.3229	6.4513	8.7648	6.8604	0.8690	0.7431	0.9853
172	cc2-185	37.7624	23.0808	6.2535	7.6650	6.7477	0.8872	0.8174	0.9850
173	cc2-186	43.1593	25.4428	5.9378	9.2468	7.2184	0.8364	0.6449	0.9855
174	cc2-187	43.3636	25.3721	6.0161	9.1094	7.0908	0.8401	0.6633	0.9841
175	cc2-188	51.0790	26.8062	7.4990	8.6403	7.8456	0.8892	0.8692	0.9863
176	cc2-189	50.4490	27.1792	6.6741	9.6186	7.4754	0.8570	0.6969	0.9862
177	cc2-190	29.7834	20.6846	5.2596	7.1939	6.0489	0.8721	0.7318	0.9843
178	cc2-191	27.2255	20.3906	4.5372	7.6168	5.3417	0.8191	0.5973	0.9811
179	cc2-192	37.0156	23.2110	5.7134	8.2170	6.6379	0.8590	0.6972	0.9836
180	cc2-193	29.2209	20.4781	5.3560	6.9383	6.4752	0.8743	0.7723	0.9844
181	cc2-195	40.5624	27.7360	6.2387	8.2641	6.8749	0.8661	0.7578	0.9851
182	cc2-196	37.0321	23.0646	5.8953	7.9862	6.3204	0.8723	0.7408	0.9834
183	cc2-197	26.1330	19.6868	4.6314	7.1591	5.5721	0.8434	0.6501	0.9818
184	cc2-198	32.2763	22.0483	5.0522	8.1170	5.5196	0.8318	0.6237	0.9834
185	cc2-199	24.8000	18.8471	4.8037	6.5431	5.6682	0.8729	0.7356	0.9823
186	cc2-200	54.2027	28.0720	7.3821	9.3276	8.7210	0.8620	0.7928	0.9862
187	cc2-201	42.9677	24.8210	6.4635	8.4509	7.1054	0.8748	0.7666	0.9844
188	cc2-202	29.9932	20.7931	5.2696	7.2276	6.0589	0.8688	0.7299	0.9824
189	cc2-203	44.0334	25.1900	6.4207	8.7190	6.7859	0.8700	0.7380	0.9860
190	cc2-204	54.8477	28.1014	7.2444	9.6084	7.9915	0.8697	0.7554	0.9855
191	cc2-205	38.4060	24.1901	5.4061	9.0260	6.3604	0.8228	0.6005	0.9833
192	cc2-206	48.7039	26.3138	6.9005	8.9566	7.2307	0.8801	0.7707	0.9863
193	cc2-207	25.0382	18.9189	4.8743	6.5268	5.3323	0.8765	0.7476	0.9824
194	cc2-208	34.7886	22.2003	5.9134	7.4633	6.6561	0.8827	0.7950	0.9845
195	cc2-209	43.2404	24.8733	6.4704	8.5041	7.3322	0.8769	0.7623	0.9857
196	cc2-210	48.3268	26.4609	6.7064	9.1512	7.2493	0.8650	0.7361	0.9856
197	cc2-211	36.1766	23.1132	5.4923	8.3549	6.3042	0.8466	0.6584	0.9846
198	cc2-212	27.1806	19.8951	4.8736	7.0771	5.9856	0.8597	0.6908	0.9830
199	cc2-213	45.7173	25.6630	6.5135	8.9173	7.1889	0.8686	0.7322	0.9857
200	cc2-214	23.5853	19.5731	4.0021	7.4742	5.3023	0.7705	0.5377	0.9791
201	cc2-215	44.4679	25.6363	6.1393	9.1705	7.2898	0.8456	0.6733	0.9848
202	cc2-216	38.5037	23.5445	6.0730	8.0686	6.5286	0.8710	0.7581	0.9844
203	cc2-218	41.9113	24.6996	6.2017	8.6060	7.2634	0.8628	0.7248	0.9845
204	cc2-219	41.7435	24.8692	5.9970	8.8532	7.1693	0.8475	0.6795	0.9846
205	cc2-220	57.5613	29.2215	7.0932	10.3142	7.8797	0.8458	0.6905	0.9848
206	cc2-221	27.4614	19.8929	5.0897	6.8615	5.6115	0.8704	0.7477	0.9829
207	cc2-222	43.9225	25.3404	6.1932	9.0022	7.1085	0.8552	0.6908	0.9846
208	cc2-223	26.3932	19.6050	4.8027	6.9671	5.5374	0.8579	0.6921	0.9825
209	cc2-224	54.3672	27.8984	7.2731	9.4928	7.8780	0.8747	0.7668	0.9866
210	cc2-225	44.8776	26.3432	5.7793	9.8646	7.1432	0.8101	0.5886	0.9845

211	cc2-226	47.2832	26.0772	6.6757	8.9916	7.4102	0.8707	0.7440	0.9857
212	cc2-227	50.6136	26.8254	7.0730	9.0883	7.0555	0.8809	0.7791	0.9861
213	cc2-228	57.7811	28.7571	7.5394	9.7177	7.4700	0.8725	0.7870	0.9857
214	cc2-229	35.0961	23.0185	5.2511	8.4870	6.3630	0.8291	0.6216	0.9840
215	cc2-230	39.9429	24.1519	5.9488	8.5325	6.6007	0.8582	0.7008	0.9854
216	cc2-231	42.5151	24.8702	6.3866	8.4582	7.4743	0.8612	0.7586	0.9861
217	cc2-232	30.7500	20.8222	5.6661	6.9029	6.1629	0.8898	0.8228	0.9837
218	cc2-233	37.9211	23.2851	6.0840	7.9031	6.7991	0.8740	0.7741	0.9843
219	cc2-234	33.5382	22.8287	4.9857	8.5610	5.8954	0.8072	0.5850	0.9824
220	cc2-236	25.7404	19.4054	4.7151	6.9316	5.6470	0.8565	0.6816	0.9821
221	cc2-237	65.2067	31.2441	7.8819	10.5286	9.1328	0.8392	0.7504	0.9869
222	cc2-238	40.2497	24.6942	5.9819	8.5526	7.4965	0.8291	0.7010	0.9839
223	cc2-239	21.7327	17.6003	4.8284	5.7198	5.2983	0.8798	0.8451	0.9826
224	cc2-241	48.0991	26.5721	6.4232	9.5030	7.9367	0.8526	0.6796	0.9861
225	cc2-242	39.2493	28.0587	5.9814	8.3300	6.4963	0.8573	0.7193	0.9852
226	cc2-243	44.1486	25.2709	6.3828	8.7973	7.2105	0.8672	0.7265	0.9851
227	cc2-244	54.6800	27.8952	7.4448	9.3157	7.7075	0.8794	0.8015	0.9863
228	cc2-245	47.4641	26.4036	6.3809	9.4385	7.3505	0.8520	0.6786	0.9854
229	cc2-246	44.9662	25.2284	6.8362	8.3658	7.2464	0.8862	0.8188	0.9848
230	cc2-247	46.0186	25.6581	6.7258	8.6746	6.7141	0.8736	0.7771	0.9842
231	cc2-248	40.4412	24.3957	5.9753	8.5884	6.6564	0.8489	0.7049	0.9836
232	cc2-249	42.3924	25.1158	5.9016	9.1169	6.7934	0.8413	0.6485	0.9852
233	cc2-250	24.2758	19.0440	4.4182	6.9648	5.6304	0.8363	0.6358	0.9816
234	cc2-251	53.5535	27.8004	7.1970	9.4537	7.6148	0.8683	0.7639	0.9853
235	cc2-253	47.1716	26.1505	6.5610	9.1467	7.1044	0.8654	0.7193	0.9857
236	cc2-254	66.8543	41.7410	7.3269	11.5928	9.2966	0.8177	0.6355	0.9871
237	cc2-255	52.2082	27.0969	7.4846	8.8503	7.8756	0.8899	0.8472	0.9867
238	cc2-256	41.0227	24.7231	5.8074	8.9815	6.8106	0.8412	0.6481	0.9845
239	cc2-257	49.3094	26.4998	7.0112	8.9430	7.6474	0.8803	0.7865	0.9862
240	cc2-258	26.2865	19.4644	4.8926	6.8260	5.5609	0.8694	0.7184	0.9830
241	cc2-259	36.1019	23.3560	5.2671	8.6672	6.1619	0.8248	0.6078	0.9839
242	cc2-260	30.4546	20.7463	5.6327	6.8739	6.3940	0.8872	0.8212	0.9835
243	cc2-261	60.5806	29.3422	7.8162	9.8490	8.2259	0.8815	0.7967	0.9870
244	cc2-262	54.3325	27.7388	7.5139	9.1933	7.8920	0.8852	0.8201	0.9869
245	cc2-263	39.7227	24.4476	5.6125	8.9813	7.0745	0.8314	0.6273	0.9852
246	cc2-264	49.8666	26.5230	7.1789	8.7900	7.8270	0.8839	0.8175	0.9861
247	cc2-265	56.1365	28.9973	6.9243	10.2881	8.3426	0.8352	0.6788	0.9848
248	cc2-267	46.7232	26.2547	6.3875	9.2944	7.3614	0.8500	0.6893	0.9844
249	cc2-269	40.7635	24.2063	6.2158	8.3237	6.9719	0.8704	0.7510	0.9856
250	cc2-270	40.8100	24.5143	6.0215	8.6258	6.6034	0.8511	0.7020	0.9832

251	cc2-271	46.5193	26.1546	6.4125	9.2308	7.4074	0.8537	0.6968	0.9848
252	cc2-272	34.4877	22.0761	5.9040	7.4047	6.4510	0.8846	0.7978	0.9842
253	cc2-273	44.0856	25.0394	6.6775	8.3734	7.2255	0.8799	0.8007	0.9851
254	cc2-274	35.3623	22.6790	5.5973	8.0318	6.5631	0.8623	0.6982	0.9841
255	cc2-275	43.4895	25.1216	6.2593	8.8289	6.8458	0.8636	0.7103	0.9850
256	cc2-276	42.8276	24.7795	6.3852	8.5194	7.3992	0.8732	0.7516	0.9857
257	cc2-277	30.2669	21.0985	5.1257	7.5089	5.8049	0.8526	0.6835	0.9816
258	cc2-278	47.4095	30.2361	6.9246	8.6887	7.0653	0.8735	0.8012	0.9860
259	cc2-279	69.1052	31.8447	8.0037	10.9678	8.7668	0.8552	0.7327	0.9859
260	cc2-280	39.4406	23.7603	6.1845	8.0849	7.0059	0.8740	0.7666	0.9845
261	cc2-281	55.9995	28.7095	7.3286	9.7190	8.8527	0.8531	0.7547	0.9865
262	cc2-282	26.8945	19.5529	5.1293	6.6488	5.8389	0.8796	0.7743	0.9822
263	cc2-283	29.2390	20.4976	5.2179	7.1122	5.6970	0.8712	0.7353	0.9830
264	cc2-284	50.8711	26.8184	7.3406	8.8093	7.8409	0.8865	0.8350	0.9858
265	cc2-285	45.4028	25.5688	6.5400	8.8314	7.2771	0.8707	0.7453	0.9855
266	cc2-286	35.8632	22.8644	5.6596	8.0546	6.5559	0.8599	0.7048	0.9838
267	cc2-287	31.2511	21.3409	5.2714	7.5328	5.8778	0.8600	0.7004	0.9821
268	cc2-288	46.3567	26.6900	5.8979	9.9919	6.9772	0.8159	0.5930	0.9851
269	cc2-289	48.3019	26.4472	6.6903	9.1779	7.4651	0.8660	0.7317	0.9852
270	cc2-290	27.9445	19.9219	5.2772	6.7308	5.6286	0.8822	0.7851	0.9829
271	cc2-291	34.7949	22.6123	5.5287	7.9966	6.1793	0.8508	0.6961	0.9830
272	cc2-292	32.1133	21.5476	5.4579	7.4827	6.4837	0.8674	0.7318	0.9834
273	cc2-293	39.7737	23.8555	6.2020	8.1447	7.2089	0.8753	0.7643	0.9850
274	cc2-294	41.1955	24.4476	6.2415	8.3533	7.1653	0.8606	0.7510	0.9824
275	cc2-295	34.6859	22.0973	5.9763	7.3698	6.1967	0.8897	0.8118	0.9851
276	cc2-296	35.9026	22.7340	5.8439	7.8162	6.3091	0.8710	0.7494	0.9837
277	cc2-297	26.8475	19.6352	4.9976	6.8253	5.5896	0.8720	0.7345	0.9823
278	cc2-298	45.0448	26.1104	5.9530	9.6173	6.9207	0.8280	0.6223	0.9852
279	cc2-299	36.4521	23.0739	6.0715	7.6378	6.6234	0.8672	0.7961	0.9823
280	cc2-300	64.1214	30.7626	7.9743	10.2208	9.2180	0.8503	0.7818	0.9864
281	cc2-301	64.3479	31.2268	7.1610	11.4280	9.2201	0.8278	0.6286	0.9861
282	cc2-302	35.4231	23.2375	5.1831	8.6716	6.2618	0.8214	0.5997	0.9842
283	cc2-303	42.8594	24.7802	6.7355	8.0922	7.3170	0.8757	0.8341	0.9863
284	cc2-304	38.9062	23.7944	5.8648	8.4031	6.8338	0.8580	0.7032	0.9843
285	cc2-306	51.2724	27.8044	6.5941	9.8813	8.5292	0.8324	0.6689	0.9871
286	cc2-307	20.3902	17.7250	4.0753	6.3523	5.2890	0.8121	0.6426	0.9791
287	cc2-308	40.3146	24.1122	6.1814	8.2972	6.7670	0.8697	0.7479	0.9850
288	cc2-309	57.3093	32.6709	7.1773	10.1383	8.2048	0.8487	0.7096	0.9854
289	cc2-310	35.8631	22.8467	5.6485	8.0666	6.8443	0.8609	0.7029	0.9845
290	cc2-311	28.8794	20.3427	5.1841	7.0643	5.7467	0.8731	0.7373	0.9830

291	cc2-312	49.7608	26.8491	6.8212	9.2637	7.7298	0.8646	0.7389	0.9859
292	cc2-313	52.8737	27.4317	7.2802	9.2081	8.0919	0.8785	0.7914	0.9861
293	cc2-315	29.1990	20.7786	4.9551	7.4858	5.6249	0.8472	0.6640	0.9823
294	cc2-317	65.7396	31.1752	7.9666	10.4828	8.6774	0.8480	0.7620	0.9846
295	cc2-318	30.4049	21.0146	5.2082	7.4145	5.4435	0.8628	0.7034	0.9827
296	cc2-319	45.7572	26.0226	6.2564	9.3029	6.9902	0.8468	0.6791	0.9853
297	cc2-320	30.9435	20.8574	5.7149	6.8761	6.2171	0.8904	0.8330	0.9843
298	cc2-321	61.2423	30.4184	7.3221	10.6212	9.0139	0.8312	0.6923	0.9862
299	cc2-323	42.5872	25.1090	6.0418	8.9616	7.1002	0.8475	0.6758	0.9839
300	cc2-324	59.7721	29.7374	7.1500	10.6302	8.1250	0.8477	0.6745	0.9868
301	cc2-325	40.2852	23.8705	6.4981	7.8794	7.1195	0.8863	0.8273	0.9852
302	cc2-326	47.3185	26.8821	6.0192	9.9747	7.0589	0.8200	0.6047	0.9849
303	cc2-327	42.8443	24.7611	6.3907	8.5145	6.9490	0.8756	0.7524	0.9854
304	cc2-328	46.3740	25.9961	6.5447	9.0151	7.4383	0.8617	0.7282	0.9851
305	cc2-329	32.9814	22.3811	5.0521	8.2971	6.4218	0.8254	0.6125	0.9839
306	cc2-330	37.8885	23.1848	6.1335	7.8478	6.3883	0.8833	0.7830	0.9854
307	cc2-331	54.7010	27.9325	7.3219	9.4999	7.9788	0.8792	0.7728	0.9869
308	cc2-332	37.0891	23.1666	5.8563	8.0594	6.4949	0.8673	0.7300	0.9849
309	cc2-333	48.7636	30.0566	7.0798	8.7389	7.5110	0.8642	0.8143	0.9837
310	cc2-334	36.8186	22.9567	5.9249	7.8959	6.5985	0.8756	0.7523	0.9844
311	cc2-335	34.9435	22.6397	5.4942	8.0855	6.0908	0.8546	0.6818	0.9843
312	cc2-336	49.8837	26.6487	7.0278	9.0083	7.6035	0.8794	0.7819	0.9862
313	cc2-337	52.4029	27.6357	6.9280	9.6184	8.0112	0.8607	0.7228	0.9852
314	cc2-338	32.7388	21.5867	5.6094	7.4007	5.9019	0.8787	0.7593	0.9837
315	cc2-339	24.9508	19.2768	4.5104	7.0170	5.8529	0.8392	0.6430	0.9818
316	cc2-340	53.0664	27.8111	6.8665	9.7980	7.4606	0.8581	0.7029	0.9867
317	cc2-341	36.3038	22.7520	5.9378	7.7610	6.0880	0.8778	0.7685	0.9839
318	cc2-342	36.5205	23.0123	6.1614	7.5457	7.0456	0.8655	0.8180	0.9840
319	cc2-343	40.4953	24.0865	6.2594	8.2153	6.8002	0.8734	0.7655	0.9849
320	cc2-344	41.4881	25.1110	5.6511	9.3141	6.6848	0.8232	0.6084	0.9851
321	cc2-345	56.0634	32.5704	7.4309	9.5836	7.9300	0.8693	0.7772	0.9869
322	cc2-346	34.4830	22.4212	5.5075	7.9345	6.5891	0.8578	0.6955	0.9842
323	cc2-347	30.8460	21.5456	5.0328	7.7875	6.7152	0.8328	0.6473	0.9848
324	cc2-348	43.2940	25.2073	6.4505	8.5282	7.4140	0.8544	0.7590	0.9823
325	cc2-349	23.8924	19.2063	4.2215	7.1881	5.4359	0.8101	0.5879	0.9806
326	cc2-350	43.7306	25.0865	6.4039	8.6784	7.2417	0.8699	0.7395	0.9855
327	cc2-351	45.0537	25.8424	6.2200	9.2171	6.9894	0.8459	0.6782	0.9845
328	cc2-352	48.1989	26.2810	6.8460	8.9371	7.2914	0.8723	0.7678	0.9858
329	cc2-353	46.8083	26.3116	6.2878	9.4510	7.2858	0.8461	0.6688	0.9852
330	cc2-354	43.1046	25.4460	5.8615	9.3359	7.2735	0.8334	0.6292	0.9848

331	cc2-355	27.7225	19.9450	5.1495	6.8454	5.9359	0.8740	0.7540	0.9820
332	cc2-356	31.5639	21.3530	5.3607	7.4690	6.4154	0.8659	0.7201	0.9842
333	cc2-357	38.6636	23.6084	5.9831	8.2202	6.9976	0.8700	0.7302	0.9853
334	cc2-358	36.4238	22.7861	5.9407	7.7936	6.3639	0.8795	0.7650	0.9846
335	cc2-359	60.4282	29.5892	7.6103	10.1033	8.0326	0.8663	0.7580	0.9850
336	cc2-360	24.5842	18.5925	5.0230	6.2170	5.6323	0.8907	0.8089	0.9828
337	cc2-361	51.8792	27.6448	6.9499	9.4782	8.0833	0.8506	0.7342	0.9868
338	cc2-362	35.3580	22.7641	5.5800	8.0548	6.7412	0.8558	0.6947	0.9833
339	cc2-363	48.4714	26.5766	6.5844	9.3546	7.3941	0.8602	0.7062	0.9857
340	cc2-364	59.6621	29.4085	7.4449	10.1821	8.2705	0.8647	0.7328	0.9869
341	cc2-365	64.1366	30.5567	7.5840	10.7364	8.4891	0.8598	0.7105	0.9879
342	cc2-366	36.6286	22.9028	5.9269	7.8526	6.7460	0.8751	0.7573	0.9847
343	cc2-367	32.2265	21.3114	5.8044	7.0494	6.1289	0.8885	0.8249	0.9841
344	cc2-368	27.9622	20.1944	4.9542	7.1627	5.8921	0.8570	0.6942	0.9824
345	cc2-369	33.5978	21.9785	5.6647	7.5351	6.4274	0.8719	0.7518	0.9839
346	cc2-370	38.1514	23.2165	6.3127	7.6825	6.4008	0.8874	0.8238	0.9849
347	cc2-371	58.6298	29.3634	7.1851	10.3718	8.2702	0.8527	0.6958	0.9868
348	cc2-372	37.5928	23.3116	5.8163	8.1905	6.9069	0.8638	0.7112	0.9846
349	cc2-373	27.9071	20.0291	5.0090	7.0422	5.6964	0.8673	0.7123	0.9817
350	cc2-374	39.4309	24.3116	5.6223	8.8914	6.3579	0.8340	0.6343	0.9842
351	cc2-375	32.4620	21.5317	5.6105	7.3549	5.6344	0.8777	0.7645	0.9831
352	cc2-376	44.6622	25.3409	6.4653	8.7805	7.6442	0.8719	0.7379	0.9855
353	cc2-377	44.0001	25.7216	5.9602	9.3955	7.1155	0.8346	0.6402	0.9843
354	cc2-378	51.9007	27.4976	6.8187	9.6662	7.3285	0.8594	0.7109	0.9862
355	cc2-380	46.8568	29.9321	6.2572	9.5164	7.6113	0.8340	0.6596	0.9852
356	cc2-381	35.5317	23.9514	4.8937	9.2460	6.1698	0.7778	0.5326	0.9824
357	cc2-383	35.0719	22.3800	5.8734	7.5950	6.5102	0.8781	0.7755	0.9848
358	cc2-384	38.7918	23.6802	5.9840	8.2443	6.4017	0.8673	0.7289	0.9843
359	cc2-385	27.3059	19.7944	5.0338	6.8722	6.0451	0.8695	0.7341	0.9829
360	cc2-386	27.2203	19.6638	5.1622	6.6847	5.8355	0.8797	0.7730	0.9822
361	cc2-387	25.8332	19.1513	5.0231	6.5311	5.1802	0.8818	0.7717	0.9813
362	cc2-388	30.1686	20.8956	5.2425	7.3141	5.9956	0.8659	0.7207	0.9834
363	cc2-389	44.6643	25.2613	6.6435	8.5393	7.3539	0.8772	0.7798	0.9853
364	cc2-390	47.1216	26.2671	6.5077	9.2023	7.2209	0.8560	0.7104	0.9843
365	cc2-391	56.2436	28.8100	6.9844	10.2203	7.9661	0.8481	0.6876	0.9858
366	cc2-392	77.1025	33.4942	8.4096	11.6539	9.3197	0.8613	0.7244	0.9885
367	cc2-393	42.0386	24.4090	6.6385	8.0163	7.2413	0.8802	0.8322	0.9838
368	cc2-394	25.4402	19.2240	4.7879	6.7502	5.3629	0.8613	0.7122	0.9816
369	cc2-395	26.0837	19.7273	4.6180	7.1690	5.4889	0.8380	0.6462	0.9821
370	cc2-396	32.8268	21.9304	5.3258	7.8264	6.0615	0.8549	0.6817	0.9839

371	cc2-397	27.9990	20.0618	5.1151	6.9566	5.5875	0.8721	0.7358	0.9828
372	cc2-399	44.2402	25.3743	6.3278	8.8782	7.1482	0.8605	0.7157	0.9852
373	cc2-400	31.2823	21.1156	5.4981	7.2241	5.9732	0.8783	0.7640	0.9839
374	cc2-401	44.6229	25.6172	6.2033	9.1382	6.9484	0.8509	0.6822	0.9851
375	cc2-402	44.6624	25.4863	6.3818	8.8966	6.9128	0.8620	0.7205	0.9841
376	cc2-403	45.2181	25.5504	6.5103	8.8302	7.5089	0.8683	0.7387	0.9857
377	cc2-404	34.4897	22.4433	5.4918	7.9714	6.3504	0.8572	0.6906	0.9845
378	cc2-405	59.6518	29.5019	7.3036	10.3623	7.9936	0.8572	0.7088	0.9867
379	cc2-406	22.7783	17.9957	4.7157	6.1375	5.1467	0.8811	0.7702	0.9818
380	cc2-407	50.0093	26.9422	6.9115	9.1773	7.7593	0.8624	0.7550	0.9843
381	DD2-001	39.1050	23.6789	6.0987	8.1422	6.5549	0.8735	0.7497	0.9848
382	DD2-002	38.3560	27.1891	6.2774	7.7648	6.5521	0.8780	0.8109	0.9852
383	DD2-003	41.4294	24.6875	5.9600	8.8247	7.0577	0.8509	0.6809	0.9854
384	DD2-006	41.8455	24.5335	6.2592	8.4929	6.9291	0.8713	0.7387	0.9850
385	DD2-008	27.4623	19.9149	4.9798	7.0007	6.0100	0.8670	0.7130	0.9835
386	DD2-009	59.9891	30.1042	7.1441	10.6609	8.4231	0.8323	0.6736	0.9843
387	DD2-010	34.5139	22.1275	5.9169	7.4156	6.5531	0.8843	0.7994	0.9844
388	DD2-011	30.1467	23.9498	5.5672	6.8809	5.8595	0.8793	0.8109	0.9836
389	DD2-012	44.9702	25.2886	6.6545	8.5782	7.1809	0.8805	0.7769	0.9858
390	DD2-013	32.9827	21.6907	5.6588	7.3971	6.3031	0.8774	0.7656	0.9839
391	DD2-014	46.1822	26.0209	6.3260	9.2653	7.6759	0.8536	0.6853	0.9858
392	DD2-016	43.9738	25.1448	6.4281	8.6903	6.8431	0.8713	0.7428	0.9860
393	DD2-017	34.1799	22.0470	5.7990	7.4818	6.5409	0.8799	0.7799	0.9843
394	DD2-018	45.0226	25.3363	6.6840	8.5638	7.1696	0.8798	0.7822	0.9857
395	DD2-019	52.0198	27.3864	6.9398	9.5054	7.5155	0.8672	0.7314	0.9856
396	DD2-020	29.4889	20.6210	5.2097	7.2012	5.9970	0.8703	0.7246	0.9834
397	DD2-021	44.8976	25.3099	6.6207	8.6120	6.9676	0.8781	0.7701	0.9858
398	DD2-022	43.4179	25.0061	6.3632	8.6736	6.8686	0.8707	0.7350	0.9859
399	DD2-023	30.6620	21.5708	4.8890	7.9755	6.2481	0.8258	0.6162	0.9829
400	DD2-024	27.1729	21.5006	4.0074	8.6220	5.6685	0.7374	0.4663	0.9809
